# Supplementary material for: Functional determinants of protein assembly into homomeric complexes
Source: Sci Rep. 2017 Jul 10;7:4932. doi: 10.1038/s41598-017-05084-8 (PMC5504011; doi:10.1038/s41598-017-05084-8)
Supplement: Supplementary file 1 — Supplementary Information [file 41598_2017_5084_MOESM1_ESM.doc]

# **Functional determinants of protein assembly into homomeric complexes**

L. Therese Bergendahl* and Joseph A. Marsh

*MRC Human Genetics Unit, Institute of Genetics and Molecular Medicine,*

*University of Edinburgh, Western General Hospital, Edinburgh EH4 2XU, UK*

**Correspondence to therese.bergendahl@igmm.ed.ac.uk*

# Supplementary Information for *“Functional determinants of protein assembly into homomeric complexes”*

## Supplementary Discussion

***Functional enrichment trends are preserved using strict filtering for redundancy at the level of domain assignments***

For the analysis in the main text of the paper, the non-redundant dataset was generated by clustering structures at the level of 50% sequence identity. However, this could possibly lead to overrepresentation of some protein families where there are multiple related structures sharing <50% sequence identity. To account for this, we generated a second non-redundant dataset where clustering was performed using SUPERFAMILY domain assignments, as described in the Methods. We then repeated our analysis for functional enrichments as in the main text but using this more strictly filtered dataset. All enrichments are presented in Table S1 and are discussed below. In general, we see the same GO terms enriched as observed using the larger dataset filtered using sequence identity, although the significance of associations is reduced due to the smaller size of the dataset.

*Twofold symmetric homomers*

The more strictly filtered dataset contains 1854 twofold symmetric homomers, compared to 4830 in the main dataset. In this smaller dataset, “*protein dimerization*” is the most significantly associated functional term (1.24-fold enrichment; *P* = 6 x 10^-6^), followed by “*carboxylic acid metabolic process* (1.23-fold enrichment; *P* = 3 x 10^-5^) and “*sequence specific DNA binding transcription factor activity*” (1.46-fold enrichment; *P* = 3 x 10^-4^). However, “*biosynthetic process*”, which is the most significantly enriched term in the main dataset, is reduced in enrichment in this dataset (1.11-fold enrichment, *P* = 0.002). This suggests that some of the enrichment of this and related terms in the main dataset is due to overrepresentation of certain related families.

*Cyclic homomers*

There are 342 cyclic structures in the more strictly filtered dataset, compared to 652 in the main dataset. Just as in the main dataset, the most significant associations are all related to transmembrane functions. For example, the most significantly enriched term is “*transmembrane transporter activity*” (4.5-fold enrichment; *P* = 6 x 10^-18^).

*Dihedral homomers*

The more strictly filtered dataset contains 692 dihedral homomers, compared to 1451 in the main dataset. Just as in the main dataset, the most significantly enriched term is “*single organism metabolic process*” (1.40-fold enrichment; *P* = 5 x 10^-17^). All of the other top enriched terms from the main dataset are also strongly enriched here.

*Cubic homomers*

There are 33 cyclic structures in the more strictly filtered dataset, compared to 82 in the main dataset. Importantly, as discussed in the main text, all but one of the ferritin structures are now excluded, yet “*cellular iron ion homeostasis*” is still the most significantly enriched term (20.6-fold enrichment; *P* = 0.004).

*Asymmetric and helical homomers*

The more strictly filtered dataset contains 236 asymmetric and helical homomers, compared to 275 in the main dataset. As discussed in the main text, these structures are problematic as a large proportion are the result of quaternary structure assignment errors. Nevertheless, the two most interesting enriched terms are also somewhat enriched in this smaller dataset: “*signal transducer activity*” (1.90-fold enrichment; *P* = 0.03) and “*DNA binding*” (1.64-fold enrichment; *P* = 0.006).

*Monomers*

There are 2274 monomers in the more strictly filtered dataset, compared to 6063 in the main dataset. The most significant positively enriched term in this dataset is “*hydrolase activity, hydrolyzing O-glycosyl compounds*” (1.38-fold enrichment; *P* = 7 x 10^-6^), which is also one of the top enriched terms in the main datset. Interestingly, “*RNA binding*” is also one of the top enriched terms (1.30-fold enrichment; *P* = 2 x 10^-5^). This further highlights our point that monomeric protein function is likely to be specialised towards large substrates, with the exception being DNA-binding, which can necessitate a symmetric binding interface such as that of dimeric homomers.

***Functional enrichment trends are preserved when controlling for monomeric structures***

The enrichment analysis in this paper is carried out by comparing the fraction of proteins from a certain symmetry group associated with a functional term to the fraction of all proteins in the dataset associated with that term. Since monomer structures comprise nearly half of our dataset, we also repeated this analysis for different symmetry groups with monomers excluded (Table S1). In general, all of the most significantly enriched terms from the main text are also highly significantly enriched. Interestingly, however, the enrichment of *“biosynthetic process”* (and related terms) in C_2_ complexes is much weaker (1.06-fold enrichment and *P* = 1 x 10^-5^; compared to 1.36-fold enrichment and *P* = 2 x 10^-61^ with monomers included). This suggests that much of the enrichment seen in C_2_ complexes is actually due to these processes being generally deficient in monomers. Importantly, however, the GO terms associated with transcription are still highly enriched in C_2_ complexes when monomers are excluded.

***Functional enrichment trends are preserved when controlling for species***

In order to address possible bias associated with the uneven distribution of species in the PDB, we also repeated our analysis for different symmetry groups using only human proteins (Table S3). As expected, the significance of any enrichment is reduced compared to the full PDB. Importantly, however, the same functionalities are enriched in the human set, where the two-fold Dimeric proteins being associated with metabolic processes and transcription, the higher order cyclic proteins being dominated by functionalities associated with the membrane, and the dihedral set of proteins highly enriched in metabolic enzyme functionalities.

## Supplementary Figures

Figure S1: The top five most significant positively enriched GO terms are tabulated with their associated P-value from Fisher’s Exact Test. (a) Homomers with cubic symmetry are dominated by functions associated with iron storage, which is partially but not completely due to the present of several ferritin complexes in our dataset. This is illustrated by the octahedral *C. tepidum* ferritin illustrated here. (b) Helical (15 total; illustrated by F-actin) and asymmetric (260 total; illustrated by homocysteine methyltransferase MmuM) are most strongly enriched in functions associated with signal transduction and DNA binding.

##

Figure S2: Enrichment of enzyme classes in monomeric proteins indicate a preference for binding to large substrates. Illustration of the enrichment of monomeric proteins within the enzyme classes defined by the Enzyme Commission. Significant negative enrichment in enzymes acting on bonds other than those involved in peptides and significant positive enrichment in glycosylases. There is also a corresponding, weak, association to enzymes acting specifically on peptide bonds. These results indicate that monomers are not associated with carbon-nitrogen bonds *unless* they are peptides. As the preferred substrates of glycolases are oligosaccharides, it appears that the enzymes in our monomer set favour large macromolecules. P-values are calculated with Fisher’s exact test and error bars represent 68% melded binomial confidence intervals.

Figure S3: Symmetry distribution in the top 16 enriched GO terms in the full set.
